# Genetic and Phenotypic Characterization of Subclinical Mastitis-Causing Multidrug-Resistant *Staphylococcus aureus*

**DOI:** 10.3390/antibiotics12091353

**Published:** 2023-08-23

**Authors:** Amanda Thaís Ferreira Silva, Juliano Leonel Gonçalves, Stéfani Thais Alves Dantas, Vera Lúcia Mores Rall, Pollyanne Raysa Fernandes de Oliveira, Marcos Veiga dos Santos, Rodolfo de Moraes Peixoto, Rinaldo Aparecido Mota

**Affiliations:** 1 Department of Veterinary Medicine, Federal Rural University of Pernambuco, Recife 52171-900, Brazil; 2 Department of Large Animal Clinical Sciences, College of Veterinary Medicine, Michigan State University, East Lansing, MI 48824, USA; 3 Department of Chemical and Biological Sciences, Institute of Biosciences, São Paulo State University, Botucatu 18618-689, Brazilvera.rall@unesp.br (V.L.M.R.); 4 Department of Animal Sciences, School of Veterinary Medicine and Animal Sciences, University of São Paulo, Pirassununga 13635-900, Brazil; 5 Federal Institute of Education, Science and Technology of Sertão Pernambucano, Petrolina 56316-686, Brazil

**Keywords:** *Staphylococcus aureus*, antibiotic resistance, intramammary infection, molecular epidemiology

## Abstract

The core objective of this study was to genetically and phenotypically characterize subclinical mastitis-causing multidrug-resistant *Staphylococcus aureus* (MDRSA). In addition, risk factors associated with subclinical mastitis caused by MDRSA were investigated. Bacterial cultures were performed on 2120 mammary quarters, 40 swabs of milk utensils, 5 bulk tank milk samples, and 11 nostril and 11 hand swabs from milkers from five dairy farms. Matrix-assisted laser desorption/ionization time-of-flight mass spectrometry (MALDI-TOF MS) was conducted for *S. aureus* identification. Antimicrobial resistance was screened phenotypically using the disk diffusion test in all *S. aureus* isolates. A biofilm formation assay; detection of genes associated with beta-lactam resistance, efflux pump, and biofilm formation; and pulsed-field gel electrophoresis (PFGE) were performed in all MDRSA isolates. Multi-locus sequence typing (MLST) was carried out in cefoxitin-resistant MDRSA isolates. A total of 188 *S. aureus* isolates from milk as well as two from milking utensils and one from bulk tank milk were identified. Most of the isolates (92.7%; 177 of 191) showed beta-lactam resistance, and 7% (14 of 191) were MDRSA. Interestingly, 36% (5 of 14) of MDRSA isolates were cefoxitin-resistant, but none carried *mecA* or *mecC* genes. Based on PFGE results, it was observed that *S. aureus* strains were more likely to be unique to a specific herd. Two clonal complexes were identified, CC97 (ST126; commonly livestock-associated) and CC1 (ST7440; usually community-associated). To the best of our knowledge, this is the first report of ST7440 isolated from bovine mastitis in Brazil. The risk factor results underscored the importance of considering parity, stage of lactation, SCC, milk production, and herd size when studying the risk of subclinical mastitis and antimicrobial resistance in *S. aureus*. Thus, to implement effective strategies to prevent subclinical mastitis in dairy herds and to minimize MDRSA spread, it is important to understand MDRSA strains’ distribution and their antimicrobial resistance profile.

## 1. Introduction

*Staphylococcus aureus* is a widespread multi-host infectious microorganism that frequently causes subclinical mastitis in Brazilian dairy herds [1] and there are many potential risk factors (e.g., age, lactation stage, milk production, herd size, milking routine, and housing management) associated with this disease [2]. In Brazil, the prevalence of *S. aureus* in dairy herds ranged from 0% to 63.3%, as reported by Dittmann et al. [3]. According to Wang et al. [4], the prevalence of *S. aureus* causing bovine mastitis varies globally, with rates ranging from 5.6% in Korea to 36.23% in China and from 46.6% to 62.4% in the United States and reaching as high as 70% in Hungary.

Recently, multidrug-resistant *S. aureus* (MDRSA) has been associated with subclinical mastitis in dairy cows and has been considered an emerging zoonotic pathogen [5]. To date, *S. aureus* can use different mechanisms of resistance to antibiotics, such as efflux-mediated resistance [6,7] and biofilm formation [6,8]. Understanding the antimicrobial resistance characteristics of *S. aureus* is critical for mastitis treatment protocols and to reduce the potential problems associated with antimicrobial resistance [9,10,11]. Several risk factors may be associated with the spread of MDRSA strains, and dairy farms can benefit from the knowledge of these factors when designing and implementing mastitis control programs [12].

To investigate the molecular epidemiology of mastitis-associated *S. aureus*, different strategies have been employed, including pulsed-field gel electrophoresis (PFGE) [13,14] and multi-locus sequence typing (MLST) [5,15]. PFGE can allow one to evaluate bacterial genetic relatedness to determine its point source during epidemiological investigations [13]. MLST makes it possible to categorize bacteria and to construct a refined and comprehensible global framework for unraveling their molecular evolution, genomic diversity, and genetic relatedness [16,17].

To the best of our knowledge, very few data are available on the presence and genetic traits of MDRSA in the milk of subclinically infected cows in the Brazilian northeast region [18,19,20,21]. Due to the current One Health concern about the emergence and spread of antimicrobial resistance of *S. aureus* in the dairy industry, the primary objective of this study was to genetically and phenotypically characterize subclinical mastitis-causing MDRSA. Another objective was to identify risk factors associated with subclinical mastitis caused by MDRSA.

## 2. Results

### 2.1. Cow Variables and S. aureus Isolates

In all dairy farms, Holstein-Gyr crossbred cattle were held in a semi-confined housing system with limited access to grazing. The mean lactating herd size was 100 cows (range of 56–229 lactating cows) and average milk production was 15 kg milk/day/cow (range of 8–36.4 kg milk/day/cow). Sample-day records included the 24 h milk yield, stage of lactation ranging from 9 to 400 days in milk (DIM), parity (primiparous or multiparous), and SCC (≤200 × 103 cells/mL and >200 × 103 cells/mL). 

In total, 2120 quarter milk samples (530 lactating cows) were examined and classified according to mastitis status (clinical, subclinical, or absent) during the study period. Overall, 201 lactating cows (37.9%) had at least one quarter with subclinical mastitis. Of the 2120 quarter observations, 801 (37.8%) had subclinical mastitis. A total of 188 *S. aureus* were recovered from milk samples of 118 lactating cows (49 from Garanhuns city, farm = 1; 10 from Águas Belas city, farm = 2; 11 from Tupanatinga city, farm = 3; and 118 from Canhotinho city, farms = 4 and 5).

Two *S. aureus* isolates were from milking utensils (farm 3 = 1; farm 4 = 1) and one from bulk tank milk (farm 4 = 1) samples. There were no *S. aureus* isolates recovered from human samples. 

Multidrug-resistant *S. aureus* (MDRSA) isolates (*n* = 14) were obtained from milk samples of 12 cows with subclinical mastitis (*n* = 12; 4 from primiparous and 8 from multiparous), bulk tank milk (*n* = 1), and milking utensils (*n* = 1).

### 2.2. Antibiotic Resistance Profile of S. aureus Isolates

Antimicrobial resistance of the *S. aureus* isolates varied from as low as 0% for nitrofurantoin to as high as 92.7% for penicillin. Penicillin was the drug with the highest resistance indexes in phenotypical analyses (92.7%), followed by Tetracycline (9.95%) and Erythromycin (6.30%; Table 1). A total of 5 out of 191 (2.62%) *S. aureus* isolates were cefoxitin-resistant. 

### 2.3. Frequency of Multidrug-Resistant S. aureus (MDRSA) Isolates

A total of 29 out of 191 *S. aureus* isolates (15.2%) showed an antimicrobial resistance profile (resistance to >1 antimicrobials) according to the multiple antimicrobial resistance (MAR) index, with 18 different phenotypes (Table 2). This profile included 3.1% (6 out of 191) of isolates that were resistant to five or more antimicrobials; 1.6% (3 out of 191) with resistance to four antimicrobials; 2.6% (5 out of 191) with resistance to three antimicrobials; and 7.9% (15 out of 191) with resistance to two antimicrobials. MDRSA was observed in 14 (7.3%) isolates. The mean MAR index for MDRSA isolates was 0.35 (range of 0.25–0.60). The most predominant MDRSA profile was penicillin, erythromycin, and clindamycin (3 out of 14 MDRSA isolates; Table 2). All cefoxitin-resistant isolates were MDRSA. 

### 2.4. Beta-Lactam Resistance, Efflux Pump, and Biofilm Formation

In our study, MDRSA isolates were obtained from four dairy farms (F1, F3, F4, and F5; Table 3). In farm 2, the lowest number of *S. aureus* isolates was recovered (*n* = 10), with no MDRSA isolates. However, Farms 4 and 5 had the highest numbers of *S. aureus* isolated (*n* = 118) and 71.4% of MDRSA isolates found (*n* = 10 out of 14). Farm 4 had the highest number of multidrug-resistant isolates (8 out of 14; 57.1%).

A total of 7 out of 14 MDRSA isolates (50%) were positive for the *blaZ* gene, which encodes beta-lactamases, and all of them were negative for *mecA* and *mecC* genes, which are inducers of the beta-lactam site of action modification. All cefoxitin-resistant MDRSA isolates were from milk samples of subclinical mastitis (5 out of 14; 35.7%), and 3 isolates (3 out of 5; 60%) were from Farm 4 (Table 3). 

A total of 12 out of 14 MDRSA isolates were obtained from primiparous (*n* = 4) and multiparous cows (*n* = 8; Table 3). Our findings revealed a higher prevalence of MDRSA isolates in multiparous cows (8 out of 12; 67%) than in primiparous cows (4 out of 12; 33%). Notably, one out of four MDRSA isolates obtained from primiparous dairy cows exhibited resistance to cefoxitin (Table 3).

Our results show that 50% (7 out of 14) of the MDRSA carried the *norA* gene, and one of those isolates also carried *norC* and *tet38* genes. None of the isolates carried the *msrA* gene (Table 3). The MDRSA isolate with the presence of the *norC* gene expressed a strong production of biofilm and, surprisingly, the same isolate did not have any assessed biofilm production gene.

All 14 MDRSA isolates were biofilm producers, classified as strong (1 out of 14; 7.15%), moderate (8 out of 14; 57.15%), and weak (5 out of 14; 35.7%) producers. A total of 86% (12 out of 14) of MDRSA isolates carried at least one biofilm formation gene, and the *icaD* gene was the most prevalent one (Table 3). In addition, 28.6% (4 out of 14) of MDRSA isolates carried the bap gene. However, none of our isolates carried the bap gene alone, bearing at least the *icaD* gene at the same time.

### 2.5. Molecular Fingerprinting of MDRSA Isolates by PFGE

**Figure 1:**
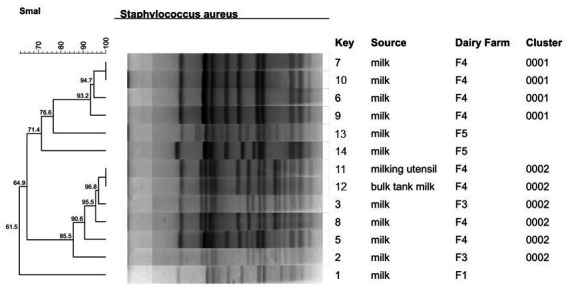
Dendrogram showing the genotypic relatedness of 13 *Staphylococcus aureus* isolates (1 MDRSA isolate was non-genotypeable; isolate 4) from different sources in four dairy herds in Pernambuco State, northeastern Brazil, which shows two clusters (1–2) at 80% similarity among the band profiles. Dendrogram was built based on the UPGMA and genetic similarity using Dice’s coefficient (1.5% tolerance) of the genotypic band patterns generated by PFGE, using the restriction enzyme *SmaI*.

The *SmaI* macrorestriction fragment profiles of 14 MDRSA isolates suggested a closely related origin and showed low genetic diversity among the isolates from four farms (F1, F3, F4, and F5). The dendrogram generated by PFGE analysis showed two different clusters (1 and 2) (Figure 1), despite one MDRSA isolate that was non-genotypeable (excluded from the analysis) and three others that were not grouped in either cluster. A total of 30.8% (4 out of 13) of the isolates were grouped in Cluster 1, and most of them (43%; 6 out of 13) were grouped in Cluster 2.

Cluster 1 had four MDRSA isolates, one cefoxitin-resistant, and all were from milk samples of multiparous cows from farm 4. A total of three out of four (75%) isolates carried the blaZ gene, and three out of four (75%) carried the *icaD* gene. All isolates formed a biofilm, 75% (three out of four) of them formed a moderate biofilm, and 25% (one out of four) of them formed a strong biofilm. Only one isolate carried efflux pump genes (*norA*, *norC*, and *tet38*).

Cluster 2 had 6 MDRSA isolates (4 from milk samples, 1 from bulk tank milk, and 1 from milk utensils) from farms 3 and 4. Two of them were cefoxitin-resistant isolates from milk samples from farm 4. Two milk samples, one from each farm, were from primiparous cows. All isolates formed a biofilm, 33.3% (two out of six) of them formed a weak biofilm, and 66.7% (four out of six) of them formed a moderate biofilm. All isolates carried at least the *icaD* gene. Four isolates carried the *norA* gene.

At a 100% similarity level, only two pulsotypes containing clonal isolates were identified, one in each cluster (1 and 2). The clonal isolates from Cluster 1 were from milk samples from Farm 4, and the clonal isolates from Cluster 2 were from milk utensils and the bulk tank milk from Farm 4.

### 2.6. Molecular Characterization of Five Cefoxitin-Resistant MDRSA Isolates by MLST

Two clonal complexes (CC) were identified, CC97 and CC1. A total of four cefoxitin-resistant MDRSA isolates (four out of five; 80%) belonged to CC97 and were assigned to ST126, and one cefoxitin-resistant MDRSA isolate (one out of five; 20%) belonged to CC1 and was assigned to ST7440.

A total of two out of the four (50%) isolates belonging to CC97 (ST126) were grouped in Cluster 1, according to the PFGE analysis (keys = 6 and 10), and both carried the beta-lactam production gene (*blaZ*) and biofilm production gene (*icaD*) and expressed moderate biofilm production. The other two isolates (two out of four; 50%) were not grouped in both clusters (keys = 1 and 13) and were biofilm producers (moderate and weak, respectively). 

The isolate belonging to CC1 (ST7440) was grouped in Cluster 2, according to PFGE analysis (Figure 1; key = 8), and carried the beta-lactam production gene (*blaZ*), efflux pump gene (*norA*), and biofilm production genes (*icaA* and *icaD*) and expressed moderate biofilm production.

### 2.7. Risk Factors 

The probability of finding lactating cows affected by MDRSA subclinical mastitis was higher in farms 4 and 5 (50% and 21.4%, respectively; *p* = 0.05), according to descriptive statistics.

According to our logistic model analysis, there was no significant difference in the odds of developing MDRSA subclinical mastitis between multiparous and primiparous cows (*p* = 0.56). However, we observed that certain risk factors (e.g., SCC, milk production, parity, and herd size) were associated with increased odds of *S. aureus* antimicrobial resistance to specific classes of antibiotics.

Cows with elevated SCC (>200 × 10^3^ cells/mL) were found to have a significantly increased risk of developing subclinical mastitis caused by beta-lactam-resistant *S. aureus* (OR = 6.4; *p* = 0.05; 95% CI = 0.96; 42.63). Additionally, within the same antibiotic class, cows with higher milk production (OR = 9.7; *p* = 0.001; 95% CI = 1.68; 56.06) and multiparous cows (OR = 4.6; *p* = 0.005; 95% CI = 0.98; 21.88) were found to be at a greater risk of developing subclinical mastitis caused by beta-lactam-resistant *S. aureus*.

Cows exhibiting elevated SCC levels showed a tendency towards developing subclinical mastitis caused by macrolide-resistant *S. aureus*. This association, although not statistically significant (*p* = 0.06), suggests a potential association between higher SCC and the occurrence of macrolide-resistant subclinical mastitis in cows. Additionally, there was a tendency observed (*p* = 0.09) where cows in smaller herd sizes (≤100 lactating cows) had greater odds of subclinical mastitis caused by tetracycline-resistant *S. aureus*.

For other antibiotic classes tested (e.g., Sulfonamide, Aminoglycoside, Nitrofuran, Fluoroquinolone, Lincosamide, and Fenicol), no associated risk factors were observed, and we believe that occurred because lower resistance levels to those antibiotics were found.

## 3. Discussion

The One Health approach is a comprehensive and collaborative strategy that recognizes the interconnectedness of human, animal, and environmental health [22]. It plays a vital role in addressing complex issues such as food safety, zoonotic diseases, and antimicrobial resistance in agriculture, including dairy production [22]. Antimicrobial resistance is an ever-evolving phenomenon that necessitates constant monitoring, and the emergence of infections caused by livestock-associated strains of MDRSA is an increasing One Health concern [10]. Developing strategies based on comprehensive information about risk factors and epidemiology is crucial to preventing MDRSA spillover to animals, humans, and the environment [2,12].

The MAR index, when it exceeds 0.2, indicates a high-risk region where antibiotics are overused [23]. Our results demonstrated a high MAR index (0.35), suggesting high antibiotic usage and, consequently, antibiotic selective pressure. In our study, only 7.3% of *S. aureus* isolates were MDRSA, but 36% of them were cefoxitin-resistant. Conversely, another Brazilian study with a similar design reported that 100% of their *S. aureus* isolates were sensitive to cefoxitin in the disk diffusion test [24]. 

According to the CLSI guideline (2018) [25], the cefoxitin disk diffusion test can be used as an alternative method of testing for methicillin-resistant *S. aureus* (MRSA). However, none of our cefoxitin-resistant MDRSA isolates carried *mecA* and/or *mecC* genes; likewise, Munive Nuñez et al. [26] did not identify either *mecA* or *mecC* resistance genes among *S. aureus* isolates from subclinical cases in Brazil. Consequently, cefoxitin resistance occurred due to other mechanisms, such as an overexpression of beta-lactamase, an uncommon phenotype like borderline-resistant oxacillin resistance, or other factors [25]. 

The bacterial efflux pump is another significant mechanism of antibiotic resistance, which is a major determinant of intrinsic and/or acquired resistance [7]. Silva et al. [27] reported the presence of *norA*, *norC*, *tet38*, and *msrA* genes in their *S. aureus* isolates. According to these results, isolates with molecular mechanisms of resistance to quinolones (e.g., *norA* and *norC* genes), tetracyclines (e.g., *tet38* gene), and macrolides (e.g., *msrA* gene) circulated in their studied farms, and these genes can play a role in multidrug resistance. Despite not having the *msrA* gene, our MDRSA isolates carried *norA*, *norC*, and *tet38* genes, which may contribute to multidrug resistance. As a first-line response to antimicrobials in staphylococci, *norA* and *norC* have a key role in efflux pump regulation [28,29]. The relative expression of *norC* is also found to be up-regulated during *S. aureus* biofilm growth [28]. The *tet38* gene can help with the extrusion of antibiotics and can also support the survival and replication of staphylococci inside the host cells [27].

Biofilms can promote bacterial tolerance to antibiotics and the transfer of resistance genes [30,31]. *S. aureus* can produce multilayered biofilm because of expression of the intercellular adhesion (*ica*) operon (e.g., *icaA* and *icaD* genes) and can adhere to surfaces, communicate cell-to-cell, and produce a biofilm without the *ica* operon due to biofilm-associated protein (*bap* gene) expression [30]. Remarkably, we detected *icaA*, *icaD*, and *bap* genes in our MDRSA isolates and they had at least a weak biofilm production, suggesting that the formation of biofilms serves as a resistance mechanism. Similar results have been reported by Marques et al. [24] and Liu et al. [32], who evaluated resistant *S. aureus* isolates from milk samples, and all of them could form biofilms. The predominantly moderate biofilm production of isolates from Farm 4 suggests it as a bacterial mechanism of persistence and transmission. It appears the pathogen has spread throughout the farm, since isolates from the same place were also recovered from subclinical mastitis cow milk, bulk tank milk, and milking utensils. 

Silva et al. [33] reported oxacillin-resistant *S. aureus* isolates from the milk of primiparous dairy cows in the northeastern region of Brazil, and most of the isolates carried the *blaZ* gene, suggesting its importance as an inducer of beta-lactam resistance. Nevertheless, none of our isolates from primiparous dairy cows carried the *blaZ* gene or efflux pump genes. There was no sampling before the first milking after parturition in our study, which means the primiparous cows may have been infected during the milking process. It underlines that *S. aureus* antibiotic resistance has many origins and several general mechanisms of adaptive responses [11]. 

Our PFGE results suggested that there is a greater likelihood of a *S. aureus* strain being unique to a specific herd than that it would appear in multiple herds at the same time, which is similar to the results reported by da Silva Soares et al. [34]. The two clonal complexes identified, CC1 and CC97, have been reported worldwide [11]. Clonal Complex (CC) 97 is a large CC, and most isolates of this lineage are animal-associated, mainly related to the dairy cattle industry [5,15,35,36,37,38]. Meanwhile, CC1 is known to contain various community-associated methicillin-resistant *S. aureus* (CA-MRSA) and is common among human isolates [36]. There is the possibility of cross-species spillover of these *S. aureus* lineages, from humans to animals and vice versa [12,39,40].

Two of the isolates from farm 4 belonged to CC97 and one to CC1, suggesting that multidrug-resistant bacteria are at high risk for spreading among cows, humans (milkers), and the environment. Given the identification of CC1, usually community-acquired multidrug-resistant staphylococcal strains, it is likely to present a substantial threat to the wellbeing of the herd. Additionally, despite CC97 being one of the lineages frequently carrying the *mecA* gene, none of our isolates in this study were identified as MRSA, despite their phenotypic resistance to cefoxitin, and similar results were previously described by Ben Said et al. [35] and Badua et al. [39]. 

The involvement of CC97 in bovine mastitis has been reported in the southeast region of Brazil [15,34,40,41]. Furthermore, CC1 has also been reported in *S. aureus* from bovine mastitis in southeastern Brazil [15,34,37,40,42]. To the best of our knowledge, there is only one report of ST126 [21] from dairy farms in northeastern Brazil, and this is the first report of the ST7440 associated with bovine mastitis in Brazil. In light of these findings, it is important to prevent MDRSA from spreading in farming areas throughout the country.

According to Silva; Laven; Benites [2], several risk factors can be associated with subclinical mastitis. In our study, the odds of *S. aureus* resistance were associated with an intrinsic and/or extrinsic risk factor depending on the antibiotic class (e.g., Beta-lactams). This result indicates the need to apply antimicrobial stewardship (AMS) efficiently in the region for optimal antimicrobial use with minimal impact on subsequent resistance. To ensure the successful implementation of AMS on dairy farms, there are several key factors, including the conscious use of antimicrobial drugs, effective communication between veterinarians and farm owners, tracking and benchmarking antimicrobial use, and educating farm workers [43].

The probability of location (*p* = 0.04) and farm (*p* = 0.05) being risk factors specific to the herd is likely influenced by the number of farms present in Canhotinho city (specifically, two farms) and the prevalence of MDRSA isolates found in those farms (71.43%, 10 out of 14). Despite implementing a strict milking order (infection-free animals first and infected animals last) and adhering to proper milking procedures, MDRSA mastitis remained a significant issue. Based on the findings from the data collection questionnaire survey and farm visits, this issue appears to stem from several factors, including inadequate maintenance of milking equipment, a lack of education about proper husbandry practices, challenging climate conditions (hot and humid), and the presence of infected and therapy-resistant cows on the farm, among other possible contributors [2,44].

Evidence indicates that horn fly (*Haematobia irritans*) is a potential vector in the transmission of *S. aureus* [45] and can be a potential risk factor associated with mastitis [46]. In our study, we collected horn flies during milking (4 horn flies from the back of 5 cows and a total of 20 flies per farm), and there was no identification of *S. aureus*. Based on our findings, it appears that the horn fly control program did not contribute as a risk factor. However, additional research is necessary to explore the potential impact of horn flies on mastitis in dairy farms.

Considering the animals, the odds of MDRSA subclinical mastitis in primiparous or multiparous lactating cows were similar. This indicates that all lactating cows were at the same risk. However, the odds of developing subclinical mastitis resistant to beta-lactam are 4.6 times greater for multiparous cows than primiparous. In contrast, Demil et al. [47] reported that multiparous cows at increased stages of lactation are at higher risk of developing subclinical mastitis despite no information being provided about the resistance of multidrug bacteria in this cited study. 

Furthermore, our risk factor results highlight the importance of considering various factors, including parity and stage of lactation, as well as SCC levels, milk production, and herd size when assessing the risk of subclinical mastitis and antimicrobial resistance in *S. aureus*. Although the sample size is limited, consisting of only 14 MDRSA strains, the dataset provides valuable insights into mastitis risk factors in an underexplored region of Brazil. However, future research should aim to include a larger number of MDRSA strains and to address the lack of accurate data from most dairy farms in the country and the challenge of engaging farm owners, which restricted the number of farms included in this study.

## 4. Materials and Methods

### 4.1. Selection of Farms and Cows

**Figure 2:**
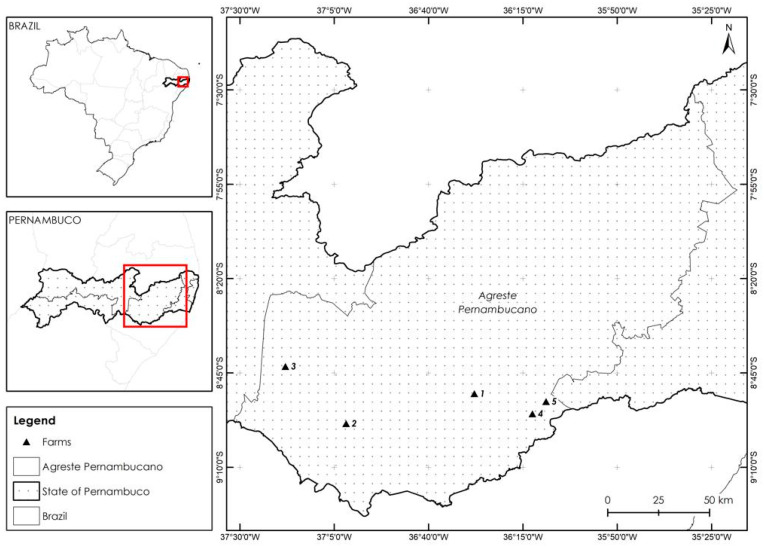
Distribution map of the farms sampled (1, 2, 3, 4, and 5), located in the Agreste region of Pernambuco State, northeastern Brazil.

A total of 5 out of 30 commercial dairy farms in the Agreste Meridional region of Pernambuco State, Brazil, were enrolled in this study (Figure 2) during a four-month sampling period (November 2020–February 2021). Farms were selected based on having an accessible recording system (e.g., herd size, stage of lactation, parity information, milk production, and monthly somatic cell count scores) and willingness to participate in this study. Farms had to have a conventional milking parlor with a mechanical milking system, perform a milking routine that includes the identification of clinical mastitis before milking (e.g., forestripping), and have cow identification (e.g., ear tags). 

At the first visit, an epidemiological questionnaire was provided to all farms about potential risk factors for mastitis (Supplementary Information). The questionnaire included questions on general farm data (e.g., farm and herd size and milk production), on individual cow details (e.g., number, age, and lactation stage), and on management measures related to mastitis (e.g., hygienic pre- and post-milking routines, use of clean gloves, cleaning and disinfecting of milking utensils, and identification and separation of cows infected with mastitis).

All lactating crossbred Holstein-Gyr cows (½ *Bos taurus taurus* × ½ *Bos taurus indicus*; *n* = 530; 200 primiparous and 330 multiparous cows) with four functional quarters and no history of clinical mastitis in the previous month of milk sampling were selected. Subclinical mastitis was confirmed based on the presence of a significant bacterial colony count (≥100 cfu/mL of *S. aureus*) in milk samples and the absence of clinical signs. 

Swabs from teat liners of the milking machine were collected at the end of milking (*n* = 40; 6 teat liners in one out of five farms, 8 teat liners in three out of five farms, and 10 teat liners in one out of five farms), and one bulk tank milk sample for each farm per visit was recovered for *S. aureus* testing. Also, nostril (*n* = 11) and hand (*n* = 11) swabs of consenting milkers (*n* = 11) on the five dairies were sampled in the intermilking period using sterile transport media swabs.

The experimental procedures were approved by the Animal Use Ethics Committee of the Federal Rural University of Pernambuco (UFRPE), Recife, Brazil (license number 5100110120) and the Research Ethics Committee of the University of Pernambuco (UPE), Recife, Brazil (CAAE number: 27859120.8.0000.5207).

### 4.2. S. aureus Identification by Matrix-Assisted Laser Desorption/Ionization Time-of-Flight Mass Spectrometry (MALDI-TOF MS)

*Staphylococcus* spp. isolates were inoculated on Blood agar plates (Becton Dickinson, Sparks, MD, USA). After a 24 h incubation, a single colony was applied to the MALDI-TOF MS steel plate spot with a disposable loop, as described by Barcelos et al. [48]. A volume of 1.0 μL of formic acid (70%) was applied to the spot and dried at room temperature. After drying, 1.0 μL of matrix solution consisting of α-cyano-4-hydroxycinnamic acid (HCCA) diluted in 50% acetonitrile and 2.5% trifluoroacetic acid were applied and again left to dry at room temperature. The plate reading was performed according to the specifications for ribosomal bacteria protein identification (Bruker Daltonik, Bremen, Germany), and spectral data processing was performed using Biotyper 3.0. A standard protein solution (Bacterial Test Standard, BTS; Bruker) was used for MALDI-TOF MS calibration. The MALDI-TOF MS analysis was performed in Microflex Bruker equipment. Data were acquired using FlexControl 3.3 software. Mass spectra were collected over a mass range of 2000 to 20,000 *m*/*z*. Three thousand laser shots were accumulated to generate each spectrum. The spectra obtained were compared with data from the MALDI Byotiper reference library. The samples of protein extracts were analyzed in automatic mode, so the Flexcontrol 3.3 software generated a fingerprint (set of protein peaks), which was used to compare with those from the reference library of the MBT 4.1.7 software containing 7311 entries. Results with scores of ≥2.0 were considered reliable for the identification of *S. aureus* at the species level.

### 4.3. Antimicrobial Susceptibility Test

*S. aureus* isolates were subjected to antimicrobial susceptibility testing with disks impregnated with 12 antimicrobial drugs: Cefoxitin (30 µg), Ceftiofur (30 µg), Clindamycin (2 μg), Chloramphenicol (30 µg), Enrofloxacin (5 μg), Erythromycin (15 µg), Gentamicin (10 μg), Nitrofurantoin (300 µg), Penicillin (10 µg), Penicillin/Novobiocin (40 µg), Tetracycline (30 µg), and Trimethoprim/Sulfamethoxazole (25μg) (Oxoid, Basingstoke, UK). In brief, the colony suspension method CLSI [25] was used to reach the 0.5 McFarland standard, the inoculum was spread on Mueller-Hinton agar supplemented with 2.0% NaCl, antibiotic disks were applied, and plates were incubated at 35 °C for 16 h. Quality control standards, the *S. aureus* ATCC^®^ 29,213 subspecies *aureus* strain, and breakpoints were used as defined by CLSI. Apparent phenotypic resistance to methicillin was considered using disk diffusion for Cefoxitin (30 µg), according to CLSI guidelines [49]. 

### 4.4. Identification of Multidrug-Resistant S. aureus (MDRSA) and Multiple Antimicrobial Resistance (MAR) Index Calculation

MDRSA was defined as resistance to at least one agent in three or more antimicrobial categories [50,51]. The MAR index was calculated and interpreted according to Krumperman [52] using the formula a/b, where ‘a’ represents the number of antibiotics to which an isolate was resistant and ‘b’ represents the total number of antibiotics tested. Fourteen *S. aureus* were considered MDRSA based on the MAR index and were used in further analyses (e.g., biofilm formation, genes detection, PFGE, and MLST). 

### 4.5. Biofilm Formation Assay

Overnight growth of MDRSA in tryptone soy broth (TSB, Difco, Leeuwarden, The Netherlands) was adjusted to the 0.5 McFarland standard, using the same broth but added by 0.5% glucose, and aliquots of 200 µL, in quadruplicate, were distributed into 96-well polystyrene microplates and incubated at 37 °C for 18 h [8]. The wells were stained with 1% crystal violet for 30 min after three washes in sterile phosphate-buffered saline (PBS). Afterwards, they were rinsed with PBS and left to dry at room temperature. Subsequently, for biofilm detachment and homogenization, 200 μL of 33% acetic acid was added to each well. An optical density (OD) of 570 nm was used to measure the wells using a microplate reader (Epoch 2 Microplate Reader, Biotek, Winooski, VT, USA). To correct the absorbance values, uninoculated wells containing tryptic soy broth were used as blanks. Based on Stépanovic et al. [53], the isolates were classified as non-biofilm producers (NP), strong biofilm producers (SBP), moderate biofilm producers (MBP), and weak biofilm producers (WBP). The OD values were previously calculated as follows: (for this type of calculation, the average OD value of the strain cannot be reduced by ODc) OD ≤ ODc = no biofilm producer; ODc < OD ≤ 2× ODc = weak biofilm producer; 2× ODc < OD ≤ 4× ODc = moderate biofilm producer; and 4× ODc < OD = strong biofilm producer. *S. aureus* ATCC^®^ 13,565 and *S. aureus* ATCC^®^ 12,600 were used as a positive and negative control, respectively.

### 4.6. Detection of Genes Associated with Beta-Lactam Resistance, Efflux Pump, and Biofilm Formation

For beta-lactam resistance, PCR was performed for the amplification of *blaZ*, *mecA,* and *mecC* genes according to the methods of Sawant et al. [54], Nakagawa et al. [55], and Paterson et al. [56], respectively (Table 4). The ATCC^®^ 29,213 *S. aureus* subspecies *aureus* strain was used as a positive control for *blaZ* gene detection, the ATCC^®^ 43,300 *S. aureus* subspecies *aureus* strain was used as a positive control for *mecA* and *mecC* detection, and DNA Free Water (QIAGEN, Hilden, Germany) was used as a negative control. For the efflux pump, PCR was performed for the detection of *msrA*, *norA*, *norC*, and *tet38* genes according to Martineau et al. [57], Truong-Bolduc et al. [58], Truong-Bolduc et al. [59], and Truong-Bolduc et al. [60] (Table 4). For biofilm formation, PCR was performed for the amplification of *bap*, *icaA*, and *icaD* genes according to the methods of Cucarella et al. [61] and Vasudevan et al. [62] (Table 4), using the Minispin kit (GE Healthcare, Little Chalfont, England) according to the manufacturer’s instructions (https://www.gelifesciences.com/gehcls_images/GELS/Related%20Content/Files/1314750913712/litdoc28916282_20161014140559.pdf (accessed on 5 July 2022). *S. aureus* ATCC^®^ 35,983 was used as a positive control for the *ica* cluster, as well as a sequenced bap-positive isolate. *Salmonella* sp. was the negative control for all PCR tests. The tests were performed using a Gene *Amp* PCR System9700 (Applied Biosystems, Carlsbad, CA, USA).

### 4.7. Pulsed-Field Gel Electrophoresis (PFGE)

Multidrug-resistant *S. aureus* isolates were fingerprinted using PFGE as described by the PulseNet protocol [63]. Briefly, each isolate was incubated in Brain Heart Infusion Broth (BHI, Difco, Leeuwarden, The Netherlands) at 37 °C for 24 h for plug preparation, using low-melting agarose and lysostaphin (100 µg/mL). After overnight incubation, approximately 2 mm of each *S. aureus* agarose plug was cut and digested with the *SmaI* Fast (Thermo Scientific, Waltham, MA, USA) restriction enzyme for 6 min. Subsequently, a single 2 mm plug of each isolate was distributed on a 1% low-melting agarose gel for electrophoresis using the CHEF Mapper at an initial switch of 5 s, final switch of 40 s, and running time of 21 h at 200 V (6 V/cm) at a temperature of 14 °C using a ramp angle of 120°. The gel was stained by using ethidium bromide (1.25 µg per mL of water; Invitrogen, Carlsbad, CA, USA) for 25 min and washed twice for 30 min with fresh distilled water. Images were taken in an image analyzer (Alphaimager—Alpha Esasy FC Software—Alphainotech Corporation, San Leandro, CA, USA) and saved as a TIFF. *S. aureus* NTCT 8325 DNA fragments were used as a base pair marker. After importing the TIFF files into the BioNumerics software (v.7.6.2, Sint-Martens-Latem), a dendrogram was generated by using the UPGMA method and the Dice coefficient to define the similarity percentage, with both tolerance and optimization set at 1.5%. Clusters were defined at 80% similarity and represented by numbers 1–2.

### 4.8. Multi-Locus Sequence Typing (MLST)

An aliquot of the pure cefoxitin-resistant MDRSA cultures (*n* = 5) was resuspended in BHI and incubated overnight at 36 °C. The PureLink™ Genomic DNA Mini Kit (Thermo-Fisher, Waltham, MA, USA) was used to purify a 1 mL aliquot of the bacterial solution. The manufacturer’s protocol for bacterial isolates was followed. MLST was performed using seven conserved housekeeping genes (*arcC*, *aroE*, *glpF*, *gmK*, *ptA*, *tpi*, and *yqiL*). The protocol of the MLST procedure, including the housekeeping gene’s amplification primers and the annealing temperatures, is available in the MLST database (http://mLst.warwick.ac.uk/mLst/dbs/Senterica (accessed on 20 December 2022). All amplifications were performed in a total volume of 50 μL per PCR reaction as described by Souza et al. [64]. A sample of the complete mix, without any DNA sample, was used as a negative control in all runs. The amplicons were sequenced by Sanger.

### 4.9. Statistical Analyses

Descriptive statistics were performed using SAS version 9.4 (SAS Institute, Cary, NC, USA), and statistical significance was defined as *p* ≤ 0.05. Descriptive statistics were used to characterize the antibiotic resistance profile of *S. aureus* isolates. PROC GLIMMIX (SAS Institute) logistic regressions were used to describe risk factors potentially associated with MDRSA or classes of antibiotics individually (beta-lactam, macrolide, tetracycline, sulfonamide, aminoglycoside, nitrofuran, fluoroquinolone, lincosamide, and fenicol) using the following dependent variables:

(1) Multidrug resistance level (binary; 0 = yes and 1 = no) and (2) classes of antibiotics individually (e.g., binary; 0 = beta-lactams and 1 = non-beta-lactams). The form of the generalized linear mixed model was:

*Y_ijklmno_* = *µ* + *herdsize_i_* + *Herd_ij_* + *SCC_k_* + *parity_l_* + *stage of lactation_m_* + *milk production_n_* + *FlyControl_o_* + *e_ijklmno_*

For antimicrobial resistance, Y was either (1) or (2). Dependent variables were binomial responses. The independent variables were fixed effects of SCC_k_ (≤200 × 10^3^ cells/mL and >200 × 10^3^ cells/mL), parity_l_ (primiparous and multiparous), stage of lactation_m_ (early <100, mid 100–200, and late >200 days), milk production_n_ (≤15 kg/day and >15 kg/day), horn fly control program (o = 2; yes and no), and herd-level variable of herd size_i_ (≤100 lactating cows and >100 lactating cows). All models included a herd (i = 5, from four different locations) nested within herd size. Backward selection was used to select variables that remained in the final models. Parity group and stage of lactation were forced in all models. The interaction factor of the parity group with the stage of lactation was also tested in previous models, but it was excluded since no significance was observed. The best models were selected, based on convergence and model fit (−2 log-likelihood and generalized chi-square/df). Estimated regression coefficients of the models were exponentiated and interpreted as odds ratios.

## 5. Conclusions

This study revealed that 7% (14 out of 191) of *S. aureus* isolates were MDRSA and 36% (5 out of 14) were cefoxitin resistant, but none of them carried *mecA* or *mecC* genes. Among the cefoxitin-resistant MDRSA isolates, two clonal complexes were identified, CC97 (ST126) and CC1 (ST7440). The presence of ST7440 in bovine mastitis in Brazil is noteworthy and, as far as we know, it has not been reported before. This study has identified some risk factors for MDRSA subclinical mastitis, but further research is required to develop targeted control programs. The evaluation of MDRSA strains’ antimicrobial resistance profiles, as well as their distribution in dairy herds, is recommended to prevent subclinical mastitis and minimize the spread of MDRSA.

## Figures and Tables

**Table 1:** Relative frequency of antimicrobial resistance of *Staphylococcus aureus* isolated from five dairy farms in Pernambuco state, Brazil, by using disk diffusion susceptibility tests.

Antibiotic Class	Antibiotic Disks (Concentration)	*S. aureus* Isolates (*n* = 191)	
		Res ^1^	RF (%) ^2^
Beta-lactam	Cefoxitin (30 µg)	5	2.62
	Penicillin (10 µg)	177	92.70
	Penicillin/Novobiocin (40 µg)	2	1.05
	Ceftiofur (30 µg)	2	1.05
Macrolide	Erythromycin (15 µg)	12	6.30
Tetracycline	Tetracycline (30 µg)	19	9.95
Sulfonamide	Trimethoprim/Sulfamethoxazole (25 μg)	2	1.05
Aminoglycoside	Gentamicin (10 μg)	7	3.7
Nitrofuran	Nitrofurantoin (300 µg)	0	0
Fluoroquinolone	Enrofloxacin (5 μg)	3	1.6
Lincosamide	Clindamycin (2 μg)	10	5.23
Fenicol	Chloramphenicol (30 µg)	2	1.05

^1^ Resistant; ^2^ Relative frequency.

**Table 2:** Resistance profile of phenotypic resistant *Staphylococcus aureus* isolates according to MAR index.

Id. ^1^	No. of Ab ^2^	No. of Isolates	Resistance Profile ^3^	Resistance to Ab Group	MAR Index
1	2	1	PEN, PNV	1	0.2
2	2	8	PEN, TET	2	0.2
3	2	2	PEN, ERY	2	0.2
4	2	1	PEN, GEN	2	0.2
5	2	2	PEN, ENO	2	0.2
6	2	1	ERI, CLI	2	0.2
7	3	1	PEN, TET, ENO	3	0.25
8	3	1	PEN, TET, CLO	3	0.25
9	3	3	PEN, ERY, CLI	3	0.25
10	4	1	PEN, ERY, CLI, GEN	4	0.3
11	4	1	PEN, ERY, CLI, TSU	4	0.3
12	4	1	PEN, ERY, CLI, TET	4	0.3
13	4	1	PEN, CFO, CFT, GEN, TSU	3	0.3
14	5	1	PEN, ERY, CLI, TET, GEN	5	0.4
15	5	1	PEN, CFO, ERY, CLI, TET	4	0.4
16	5	1	PEN, PNV, CFO, ERY, CLI	3	0.4
17	6	1	PEN, CFO, ERY, CLI, TET, GEN	5	0.5
18	7	1	PEN, CFO, CFT, ERY, CLI, TET, GEN	5	0.6

^1^ Identification of the phenotypes; ^2^ Number of antibiotics; ^3^ CFO—Cefoxitin, CFT—Ceftiofur, CLI—Clindamycin, CLO—Chloramphenicol, ENO—Enrofloxacin, ERY—Erythromycin, GEN—Gentamicin, PEN—Penicillin, PNV—Penicillin/Novobiocin, TET—Tetracycline, TSU—Trimethoprim/Sulfamethoxazole.

**Table 3:** Characteristics of the 14 multidrug-resistant *Staphylococcus aureus* strains recovered from bovine milk, bulk tank milk, and milking utensils.

Farm	ID	Source	Lactation	Phenotypic Resistance *	Βeta-Lactam Resistance Genes	Efflux Pump Genes	Biofilm Production Genes	Biofilm Formation on Polystyrene (570 nm)	PFGE Cluster
F1	1	Milk	1	PEN, CFO, CFT, GEN, TSU	*-*	*-*	*-*	Moderate	-
F3	2	Milk	1	PEN, ERY, CLI	*-*	*-*	*icaA, icaD*	Weak	2
F3	3	Milk	3	PEN, ERY, CLI	*blaZ*	*norA*	*icaA, icaD*	Weak	2
F3	4	Milk	2	PEN, ERY, CLI	*-*	*norA*	*icaD*	Weak	non-genotypeable
F4	5	Milk	1	PEN, TET, ENO	*-*	*-*	*icaD, bap*	Moderate	2
F4	6	Milk	2	PEN, CFO, ERY, CLI, TET, GEN	*blaZ*	*-*	*icaA, icaD, bap*	Moderate	1
F4	7	Milk	2	PEN, ERY, CLI, GEN	*-*	*-*	*icaA, icaD, bap*	Moderate	1
F4	8	Milk	4	PEN, CFO, ERY, CLI, TET	*blaZ*	*norA*	*icaA, icaD*	Moderate	2
F4	9	Milk	4	PEN, TET, CLO	*blaZ*	*norA, norC, tet38*	*-*	Strong	1
F4	10	Milk	2	PEN, CFO, CFT, ERI, CLI, TET, GEN	*blaZ*	*-*	*icaD*	Moderate	1
F4	11	Milking utensil	N/A	PEN, ERY, CLI, TET	*blaZ*	*norA*	*icaA, icaD*	Moderate	2
F4	12	Bulk tank milk	N/A	PEN, ERY, CLI, TET, GEN	*-*	*norA*	*icaD*	Moderate	2
F5	13	Milk	2	PEN, PNV, CFO, ERY, CLI	*blaZ*	*norA*	*icaD, bap*	Weak	-
F5	14	Milk	1	PEN, ERY, CLI, TSU	*-*	*-*	*icaD*	Weak	-

N/A: not applicable, * CFO—Cefoxitin (30 µg), CFT—Ceftiofur (30 µg), CLI—Clindamycin (2 μg), CLO—Chloramphenicol (30 µg), ENO—Enrofloxacin (5 μg), ERY—Erythromycin (15 µg), GEN—Gentamicin (10 μg), PEN—Penicillin (10 µg), PNV—Penicillin/Novobiocin (40 µg), TET—Tetracycline (30 µg), TSU—Trimethoprim/Sulfamethoxazole (25 μg).

**Table 4:** Genes, oligonucleotide sequences, size of amplified fragments, and reference.

Gene	Primer	Sequence (5′–3′)	Amplicon Size (bp)	Reference
*blaZ*	blaZ-F blaZ-R	AAG AGA TTT GCC TAT GCT TC GGC AAT ATG ATC AAG ATA C	517	[54]
*mecA*	mecA-F mecA-R	TGG TAT GTG GAA GTT AGA TTG GGA T CTA ATC TCA TAT GTG TTC CTG TAT TGG C	155	[55]
*mecC*	mecC-F mecC-R	CAT TAA AAT CAG AGC GAG GC TGG CTG AAC CCA TTT TTG AT	188	[56]
*msrA*	msrA-F msrA-R	TCC AAT CAT TGC ACA AAA TC AAT TCC CTC TAT TTG GTG GT	890	[57]
*norA*	norA-F norA-R	TGC AAT TTC ATA TGA TCA ATC CC AGATTGCAATTCATGCTAAATATT	150	[58]
*norC*	norC-F norC-R	ATA AAT ACC TGA AGC AAC GCC AAC AAA TGG TTC TAA GCG ACC AA	200	[59]
*tet38*	tet38-F tet38-R	TTC AGT TTG GTT ATA GAC AA CGT AGA AAT AAA TCC ACC TG	200	[60]
*bap*	bap-F bap-R	CCC TAT ATC GAA GGT GTA GAA TTG GCT GTT GAA GTT AAT ACT GTA CCT GC	97	[61]
*icaA*	icaA-F icaA-R	CCT AAC TAA CGA AGG TAG AAG ATA TAG CGA TAA GTG C	1315	[62]
*icaD*	icaD-F icaD-R	AAA CGT AAG AGA GGT GG GGC AAT ATG ATC AAG ATA C	381	[62]

## Data Availability

All relevant data are within the manuscript.

## Supplementary Materials

The following supporting information can be downloaded at: https://www.mdpi.com/article/10.3390/antibiotics12091353/s1.
