# Supplementary material for: Genetic and Phenotypic Characterization of Subclinical Mastitis-Causing Multidrug-Resistant Staphylococcus aureus
Source: Antibiotics (Basel). 2023 Aug 23;12(9):1353. doi: 10.3390/antibiotics12091353 (PMC10525230; doi:10.3390/antibiotics12091353)
Supplement: Supplementary file 1 [file antibiotics-12-01353-s001.zip › antibiotics-2542757-supplementary.pdf]

Genetic and Phenotypic Characterization of Subclinical Mastitis-Causing Multidrug-Resistant *Staphylococcus aureus*

General Data
Date:
Address:
Farm name:
Farmer name:
Contact:
E-mail:

Farm Information
1. Is the property computerized?
2. Type of system:
- Grazing
- Semiconfined
- Confined
- Other
3. Production Model:
- Conventional
- Transition to organic
- Organic

Herd Information
1. Number of animals
2. Number of lactating cows
3. Number of primiparous lactating cows
4. Number of multiparous lactating cows
5. Number of dry cows

Reproduction Information
1. Breeding System
- Artificial insemination
- Use of bull
- Transfer of embryos
2. Racial characteristic:
- Purebred
- Crossbred
3. Main breed on the farm:
- Holstein
- Jersey
- Crossbreed Holstein X Gyr
- Others

Milk Production Information
1. Total milk production (liters/month)
2. Average productivity of cows (liters/cow)
3. Average milk price (R$/liter)
4. Bonus on the value of milk (R$/liter)
5. Milk quality in the last month (tank milk):
- Somatic cell count (SCC)
- Total bacterial count in milk (TBC)
6. Individual Somatic cell count (SCC)

Mastitis Information
1. Perform treatment of cases with:
- Clinical Mastitis
- Subclinical Mastitis
- Variable
2. Which medications utilize to treat mastitis:
- Antibiotic tube
- Systemic Antibiotic
- Anti-inflammatory
- Dry cow antibiotic
- Homeopathy
- Others
3. Do you do antimicrobial rotation?
4. Did you treat subclinical mastitis?
5. Value in milk price penalty for high CCS level

Preventive Measures Information
1. What are the preventive measures used in the property?
- Milking order (infection-free animals first; infected animals last)
- *Pre-dipping*
- *Post-dipping*
- Wash dirty teat
- Wash dirty udder
- Wash milking liners between milking
- Wash milking liners end milking
- Use of separate cloth or paper
- Clean milking parlor
- Milking Maintenance

- Gloves
- Dry cow treatment
- Vaccine
- Protocol treatment by veterinary
- Keep cows standing after milking
- Horn fly control program
- Training personnel regularly
- Environmental management (animal population density, floor, bedding, ventilation)
- Others

2. What mastitis control measures are being used on the property:
- Antibiogram and culture.
- Somatic cells account (SCC), bacterial total account (BTC), and tank milk composition analysis
- Individual SCC
- California mastitis test (CMT)

3. What mastitis control measures frequency (weekly, biweekly, or monthly)?

Milking Routine Information

1. Describe the milking parlor as to cleanness, type of floor, and cover.
2. How many clusters of liners are used in the milking.
3. Number of milking/day.
4. Number of cows milked Simultaneously.
